# Supplementary material for: A Novel Small Molecular Prostaglandin Receptor EP4 Antagonist, L001, Suppresses Pancreatic Cancer Metastasis
Source: Molecules. 2022 Feb 11;27(4):1209. doi: 10.3390/molecules27041209 (PMC8879074; doi:10.3390/molecules27041209)
Supplement: Supplementary file 1 [file molecules-27-01209-s001.zip › molecules-1584823-supplementary.pdf]

molecules

MDPI

Supplementary Material

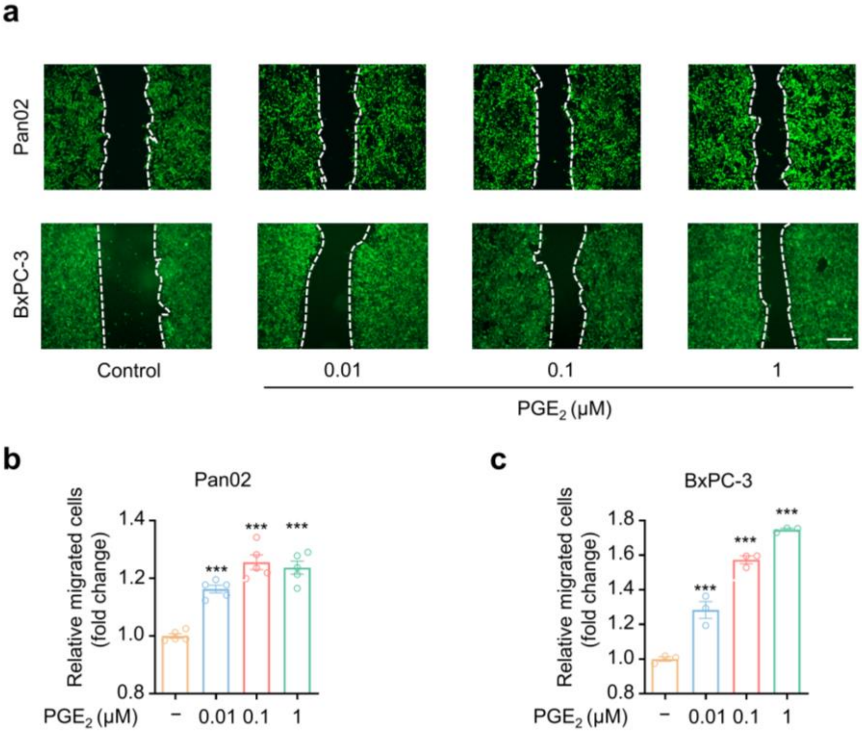

Figure S1. PGE_2 promotes pancreatic cancer cell migration. (a) Cell motility analysis of Pan02 and BxPC-3 cells treated with indicating concentrations of PGE_2 for 16 hours via wound healing assay. Scale bar, 200 μm; (b-c) Quantification of cell motility of Pan02 (b) and BxPC-3 (c) cells treated with indicating concentrations of PGE_2 as in (a) (n=3). The P value was calculated by one-way ANOVA with multiple comparison test. *** indicates P < 0.001. All the data are presented as mean ± SEM.

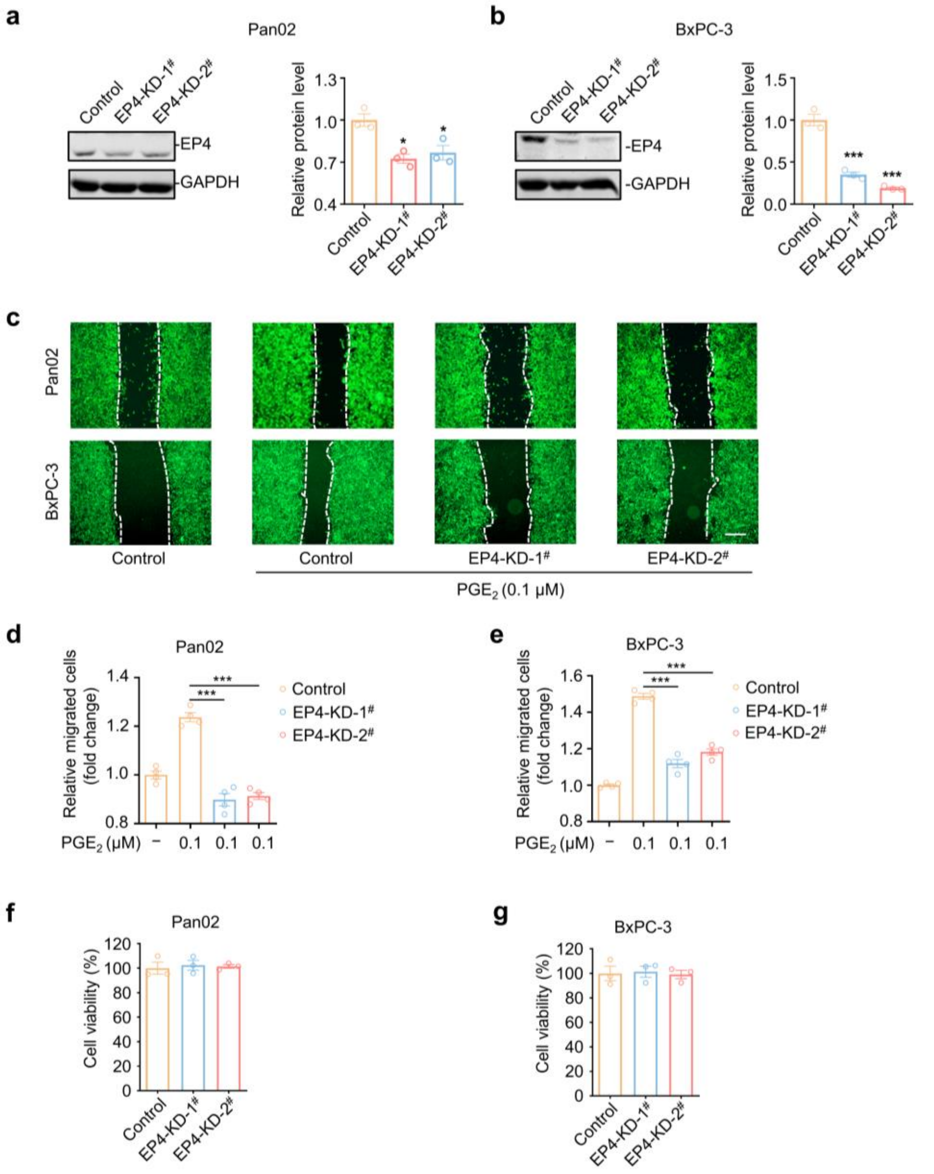

Figure S2. EP4 is essential for PGE_2-induced pancreatic cancer cell migration. (a-b) Immunodetection of EP4 expression in EP4-knockdown Pan02 (A) and BxPC-3 (B) cells. GAPDH was used as a loading marker; (c) Cell motility analysis of Pan02 and BxPC-3 cells transfected with a scramble shRNA (Control) or shRNAs targeting EP4 (EP4-KD), followed by a 24-hour treatment with DMSO or 0.1 μM PGE_2 via wound healing assay. Scale bar, 200 μm; (d-e) Quantification of cell motility of Pan02 (d) and BxPC-3 (e) cells in (c) (n = 4); (f-g) Cell viability of EP4-knockdown Pan02 (f) and BxPC-3 (g) cells (n = 3). The P value was calculated by one-way ANOVA with multiple comparison test. * indicates $P < 0.05$; *** indicates $P < 0.001$. All the data are presented as mean ± SEM.

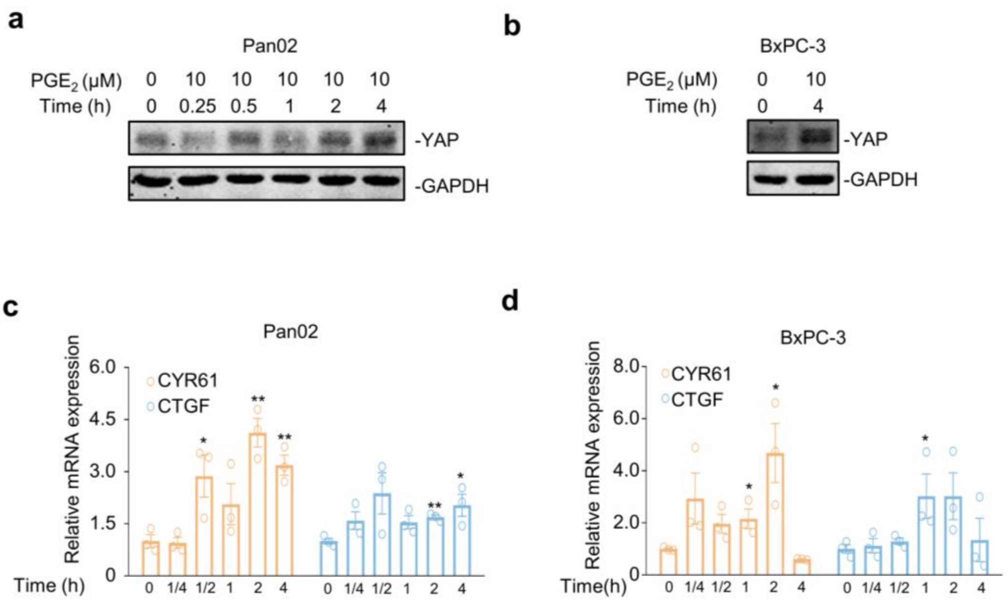

Figure S3. PGE_2 activates Hippo–YAP pathway in pancreatic cancer cells. (a-b) Immunodetection of YAP expression in Pan02 (a) and BxPC-3 (b) cells treated with DMSO or 10 μM PGE_2 for indicating times. GAPDH was used as a loading marker; (c-d) Boxplots of relative mRNA expressions of CYR61 and CTGF in Pan02 (c) and BxPC-3 (d) cells treated with 10 μM PGE_2 for indicating times. mRNA levels were determined by qPCR (n = 3). The P value was calculated by one-way ANOVA with multiple comparison test. * indicates $P < 0.05$; ** indicates $P < 0.01$. All the data are presented as mean ± SEM.

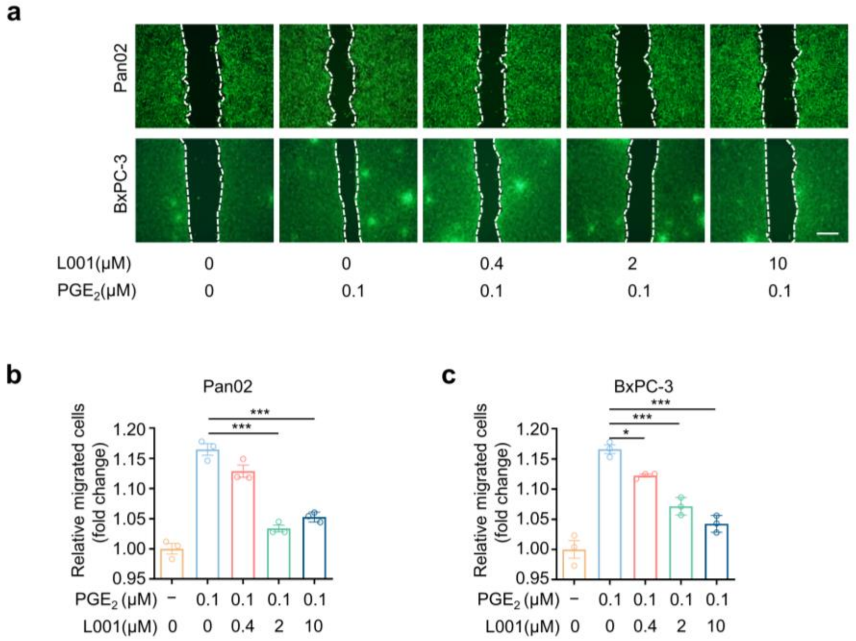

Figure S4. L001 impairs pancreatic cancer migration and invasion in vitro. (a) Cell motility analysis of Pan02 and BxPC-3 cells treated with indicated concentrations of L001 and PGE_2 for 24 hours via wound healing assay. Scale bar, 200 μm; (b-c) Quantification of cell motility of Pan02 (b) and BxPC-3 (c) cells in (a) (n = 3). The P value was calculated by one-way ANOVA with multiple comparison test. * indicates $P < 0.05$; *** indicates $P < 0.001$. All the data are presented as mean ± SEM.

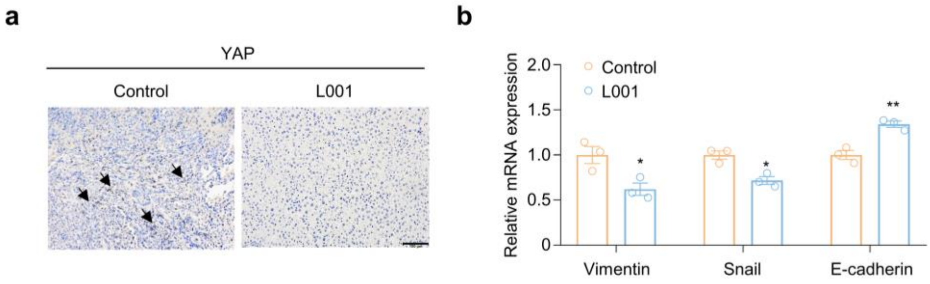

Figure S5. L001 treatment impairs YAP expression in hepatic metastases of pancreatic cancer in vivo. (a) Representative IHC immunostaining images for YAP expression of hepatic metastatic pancreatic cancer tissues from mice of Pan02-Luc pancreatic cancer metastasis model. Scale bar = 250 μM; (b) Boxplots of relative mRNA expressions of Vimentin, Snail and E-cadherin in hepatic tumor tissues of Pan02 pancreatic cancer metastasis model. mRNA levels were determined by qPCR (n = 3). The P value was calculated by two-tailed unpaired Student's t test. * indicates $P < 0.05$; ** indicates $P < 0.01$. All the data are presented as mean ± SEM.

Table S1. The primer sequences of PCR.

Symbol	Direction	Primer sequences (5′-3′)
ACTB	Forward	GTACGCCAACACAGTGCTG
	Reverse	CGTCATACTCCTGCTTGCTG
Mouse Vimentin	Forward	ATGCCGCCGCTCTAATACTT
	Reverse	GGAAAGGCCCAAGCCATCAT
Mouse E-cadherin	Forward	GGGACTGATGCTGGTGACAA
	Reverse	ACAGGTCTGTTGGGAGTGGT
Mouse Snail	Forward	CCATCCAGAGCTTGACGGTG
	Reverse	TGGGGGTTGAGGCAAACTTC
Human CYR61	Forward	AGCCTCGCATCCTATACAACC
	Reverse	TTCTTTCACAAGGCGGCACTC
Mouse CYR61	Forward	TCAGTCAGAAGGCAGACCCT
	Reverse	GGGTTGAAAAGAACTCGCGG
Human CTGF	Forward	AAAAGTGCATCCGTACTCCCA
	Reverse	CCGTCGGTACATACTCCACAG
Mouse CTGF	Forward	GCATCTCCACCCGAGTTACC
	Reverse	TCTGTGCACACCCCGCAG
Human YAP	Forward	TGGGAGATGGCAAAGACATCTTCTG
	Reverse	ACACTGGATTTTGAGTCCCACCATC
Mouse YAP	Forward	AGGAGAGACTGCGGTTGAAA
	Reverse	CCCAGGAGAAGACACTGCAT
Human EP4	Forward	CAGCACTGCTCAGACAGTCA
	Reverse	GTGAGGTGGTGTCTTCCTGG
Mouse EP4	Forward	TTTCTTCGGTCTGTCGGGTC
	Reverse	GGCTGTAGAAGTAGGCGTGG

Table S2. The Ct value of qPCR.

Figure	Target gene	Group description	Replicate 1	Replicate 2	Replicate 3
**Figure 2e**	YAP	Control	23.985	23.862	24.171
		EP4-OE	22.458	22.519	22.891
		Control	24.344	24.253	24.079
		EP4-KD	25.962	26.585	26.245
	CYR61	Control	23.228	23.234	23.082
		EP4-OE	22.166	22.194	22.294
		Control	23.663	23.389	24.090
		EP4-KD	25.630	26.207	26.212
	CTGF	Control	23.580	23.824	24.221
		EP4-OE	22.143	22.466	22.721
		Control	23.734	23.774	24.032
		EP4-KD	25.538	26.048	25.338
	ACTB	Control	16.708	16.918	16.646
		EP4-OE	16.228	16.035	16.245
		Control	16.450	16.319	16.472
		EP4-KD	17.565	17.786	17.612
**Figure 2f**	YAP	Control	23.215	22.001	22.375
		EP4-OE	22.350	22.429	22.753
		Control	22.524	22.641	22.886
		EP4-KD	24.770	24.021	24.328
	CYR61	Control	21.598	21.151	21.935
		EP4-OE	21.563	21.157	21.905
		Control	21.864	22.234	22.648
		EP4-KD	24.696	24.748	23.325
	CTGF	Control	26.209	25.701	25.964
		EP4-OE	26.208	25.563	26.197
		Control	26.289	25.977	26.410
		EP4-KD	28.564	27.928	27.133
	ACTB	Control	14.653	14.126	14.470
		EP4-OE	14.948	14.813	15.133
		Control	14.102	14.284	14.479
		EP4-KD	15.942	15.550	15.446
**Figure 5c**	YAP	Control	24.669	24.722	24.208
		PGE2	23.878	23.544	24.414
		PGE2+E7046	24.924	24.816	24.751
		PGE2+L001	24.623	24.360	24.487
	CYR61	Control	23.352	23.145	22.525
		PGE2	21.614	21.169	22.112
		PGE2+E7046	23.265	23.364	23.125
		PGE2+L001	22.794	22.562	22.655
	CTGF	Control	24.047	23.096	23.464
		PGE2	20.809	20.389	20.652
		PGE2+E7046	22.133	22.124	21.699
		PGE2+L001	21.568	21.635	21.394

	ACTB	Control		16.039	15.764	15.430
		PGE2		15.383	15.163	15.790
		PGE2+E7046		15.856	15.627	15.702
		PGE2+L001		15.370	15.272	15.247
**Figure 5d**	YAP	Control		25.478	24.117	24.076
		PGE2		22.457	22.582	22.809
		PGE2+E7046		23.426	23.807	23.830
		PGE2+L001		24.826	24.677	24.371
	CYR61	Control		24.486	23.846	23.986
		PGE2		20.080	20.666	20.648
		PGE2+E7046		20.885	20.743	20.826
		PGE2+L001		21.979	22.392	21.587
	CTGF	Control		29.195	29.159	28.932
		PGE2		25.789	26.582	26.688
		PGE2+E7046		25.660	27.142	27.842
		PGE2+L001		28.195	28.465	28.674
	ACTB	Control		17.014	16.701	16.638
		PGE2		16.834	16.972	17.397
		PGE2+E7046		17.194	17.296	17.509
		PGE2+L001		17.304	17.438	17.527
**Figure 5e**	Vimentin	PGE2 (-)	DMSO	25.248	24.809	25.123
		PGE2 (0.1 μM)	DMSO	24.036	24.020	24.413
		PGE2 (0.1 μM)	L001 (2 μM)	25.112	24.542	25.178
		PGE2 (0.1 μM)	L001 (10 μM)	24.799	24.767	25.307
		PGE2 (0.1 μM)	E7046	24.975	24.785	25.098
**Figure 5f**	Snail	PGE2 (-)	DMSO	27.234	26.749	27.180
		PGE2 (0.1 μM)	DMSO	26.421	26.261	26.923
		PGE2 (0.1 μM)	L001 (2 μM)	27.111	26.786	27.486
		PGE2 (0.1 μM)	L001 (10 μM)	27.610	27.232	27.989
		PGE2 (0.1 μM)	E7046	26.885	27.262	27.277
**Figure 5g**	E-cadherin	PGE2 (-)	DMSO	21.630	21.462	21.548
		PGE2 (0.1 μM)	DMSO	21.564	21.650	22.068
		PGE2 (0.1 μM)	L001 (2 μM)	21.876	21.422	22.283
		PGE2 (0.1 μM)	L001 (10 μM)	21.334	21.375	21.577
		PGE2 (0.1 μM)	E7046	21.930	22.177	22.245
**Figure 5e-g**	ACTB	PGE2 (-)	DMSO	15.244	14.982	15.291
		PGE2 (0.1 μM)	DMSO	14.964	15.051	15.432
		PGE2 (0.1 μM)	L001 (2 μM)	15.716	15.223	15.783
		PGE2 (0.1 μM)	L001 (10 μM)	15.248	15.299	15.701
		PGE2 (0.1 μM)	E7046	15.384	15.667	15.839
**Figure S3c**	CYR61	0 (h)		24.826	25.296	25.126
		1/4 (h)		24.298	24.929	25.207
		1/2 (h)		22.811	23.639	22.414
		1 (h)		24.334	23.650	22.579
		2 (h)		21.928	22.645	22.002
		4 (h)		22.328	23.007	22.475
	CTGF	0 (h)		24.631	24.373	24.084

		1/4 (h)	22.889	23.702	23.572
		1/2 (h)	22.251	23.425	22.079
		1 (h)	23.081	23.182	23.241
		2 (h)	22.667	22.885	22.804
		4 (h)	22.452	23.235	22.098
	ACTB	0 (h)	17.477	17.059	17.177
		1/4 (h)	16.835	16.886	17.001
		1/2 (h)	16.788	16.576	16.439
		1 (h)	16.902	16.600	16.474
		2 (h)	16.206	16.602	16.466
		4 (h)	16.426	16.650	16.325
**Figure S3d**	CYR61	0 (h)	23.883	23.963	24.210
		1/4 (h)	22.684	21.602	23.156
		1/2 (h)	22.676	22.832	22.787
		1 (h)	22.198	22.683	22.515
		2 (h)	21.123	21.261	21.812
		4 (h)	23.746	24.322	24.167
	CTGF	0 (h)	28.594	28.532	29.521
		1/4 (h)	27.810	29.213	28.871
		1/2 (h)	28.796	28.157	27.510
		1 (h)	27.489	27.031	26.064
		2 (h)	26.420	26.960	27.261
		4 (h)	29.321	26.877	28.759
	ACTB	0 (h)	16.079	16.320	16.419
		1/4 (h)	15.956	16.150	16.333
		1/2 (h)	16.340	15.957	15.544
		1 (h)	15.978	15.645	15.740
		2 (h)	16.104	15.763	15.495
		4 (h)	15.282	15.895	15.639
**Figure S5b**	Vimentin	Control	22.926	23.406	24.024
		L001	25.584	24.760	25.477
	Snail	Control	25.064	25.195	25.484
		L001	26.976	26.482	27.114
	E-cadherin	Control	20.221	20.416	20.599
		L001	21.282	20.853	21.214
	ACTB	Control	15.128	15.466	15.753
		L001	16.670	16.363	16.703

Table S3. The sequences of shRNA.

Name	Primer sequences (5′-3′)
Human EP4 shRNA-1#	CTCGAGATCTTCTCTATGGCTTTACTGTTCAAGAGACAGTAAAGCCA-TAGAGAAGATTTTTTTAAGCTT
Human EP4 shRNA-2#	CTCGAGCCAGTGAAACTCTGAAATTATTTCAAGAGAATAATTTCAGAG-TTTCACTGGTTTTTTAAGCTT
Mouse EP4 shRNA-1#	CTCGAGATAAGGGTCCAGAAACAGTACTTCAAGAGAGTACTGTTTCTG-GACCCTTATTTTTTTAAGCTT
Mouse EP4 shRNA-2#	CTCGAGATCTTCTCTATTGCTTTACTGTTCAAGAGACAGTAAAGCAA-TAGAGAAGATTTTTTTAAGCTT
